# Fourier-transform infrared spectroscopy for monitoring proteolytic reactions using dry-films treated with trifluoroacetic acid

**DOI:** 10.1038/s41598-020-64583-3

**Published:** 2020-05-12

**Authors:** Kenneth Aase Kristoffersen, Aart van Amerongen, Ulrike Böcker, Diana Lindberg, Sileshi Gizachew Wubshet, Heleen de Vogel-van den Bosch, Svein Jarle Horn, Nils Kristian Afseth

**Affiliations:** 1 Nofima - Norwegian Institute of Food, Fisheries and Aquaculture Research, P.O. Box 210 N-1431, Ås, Norway; 2 Faculty of Chemistry, Biotechnology and Food Science, Norwegian University of Life Sciences (NMBU), P.O. Box 5003, N-1432 Ås, Norway; 3 BioSensing & Diagnostics, Wageningen Food & Biobased Research, Wageningen University & Research, Bornse Weilanden 9, 6708 WG Wageningen, the Netherlands

**Keywords:** Biotechnology, Chemistry

## Abstract

In this study we explore the potential of using Fourier-transform infrared (FTIR) spectra of trifluoroacetate-protein and peptide complexes for monitoring proteolytic reactions. The idea of treating dry-films of protein hydrolysates with trifluoroacetic acid (TFA) prior to FTIR analysis is based on the unique properties of TFA. By adding a large excess of TFA to protein hydrolysate samples, the possible protonation sites of the proteins and peptides will be saturated. In addition, TFA has a low boiling point when protonated as well as complex-forming abilities. When forming TFA-treated dry-films of protein hydrolysates, the excess TFA will evaporate and the deprotonated acid (CF_3_COO^−^) will interact as a counter ion with the positive charges on the sample materials. In the study, spectral changes in TFA-treated dry-films of protein hydrolysates from a pure protein and poultry by-products, were compared to the FTIR fingerprints of untreated dry-films. The results show that time-dependent information related to proteolytic reactions and, consequently, on the characteristics of the protein hydrolysates can be obtained. With additional developments, FTIR on dry-films treated with TFA may be regarded as a potential future tool for the analysis of all types of proteolytic reactions in the laboratory as well as in industry.

## Introduction

Fourier-transform infrared (FTIR) spectroscopy is among the well-established methods for the characterisation of proteins and peptides. The repeated amino acid building blocks constructing the backbone of proteins and peptides give rise to multiple distinctive infrared absorption bands (i.e. the amide bands), containing both chemical and structural information. FTIR is therefore frequently used to study and quantify secondary structures of proteins^1–3^. At the same time, the protein information in the FTIR spectra has opened the possibility to study a wide range of protein-derived quality features. This includes parameters such as hydration and solvent effects, pH, peptide size distribution and degree of hydrolysis (DH%)^4–9^. Currently, peptide size distribution and DH% are measured using laborious and time-consuming techniques. FTIR, on the other hand, represents a rapid alternative which is applicable to an industrial setting. Recent studies have shown that FTIR spectroscopy can be used to predict parameters such as weight-average molecular weight (*M*_w_) derived from the peptide size distribution and DH% with high accuracy^10–12^.

In recent years, enzymatic protein hydrolysis (EPH) has gained significant attention as a versatile processing technology for protein-rich raw materials. For instance, several value-added peptide products have been developed employing EPH on food-processing by-products. In EPH, proteolytic enzymes are used to break down the proteins into smaller fragments, and the resulting protein hydrolysates are very complex, consisting of thousands of different protein fragments and peptides. Protein hydrolysates originating from different raw materials, such as by-products from poultry and fish production, and even different enzymes will display unique FTIR fingerprints^10^. The fingerprint differences are linked to the raw material composition and the proteolytic enzyme mode of action, giving rise to distinct protein degradation patterns, observed in the FTIR spectra. The authors recently reported a generic FTIR-based method for monitoring the weight-average molecular weights of protein hydrolysates during enzymatic hydrolysis of by-products from the food industry^10^. In that study, the best predictions were obtained using a hierarchical regression approach. The method involved supervised classification of the FTIR spectra according to raw material quality and the enzyme used in the hydrolysis process, and subsequent local regression models tuned to specific enzyme-raw material combinations. In other words, if different raw material and enzyme combinations are used, separate models for each combination are usually required. This confirms the predictive ability of the FTIR model towards protein size distribution and DH% is indeed raw material specific. This has also several practical implications, such as, needing larger spectral libraries when the aim is to establish industrially feasible generic prediction models for weight-average molecular weights of protein hydrolysates. A natural question that arises from the previous study is, therefore, if there are ways to reduce the raw material specific information in the FTIR spectra, while keeping the information related to peptide size distributions.

Trifluoroacetic acid (TFA) is frequently used for the denaturation, precipitation and analysis of proteins and peptides in biological samples. Additionally, TFA is known for its ability to interact with, and modify, the structure of proteins and peptides^13–15^. TFA is often used in the synthesis and analysis of peptides and proteins and is, therefore, a common contaminant. This can be an issue in many applications, e.g. when used in biological assays^16,17^. As a result of the extensive use of TFA in many techniques, multiple methods for removal of TFA exist, ranging from acid treatment to chromatographic methods^18,19^. When TFA interacts with proteins and peptides, complexes are formed that can be dried into films^13,20^. Such trifluoroacetate-protein and peptide complexes have previously been characterised using FTIR spectroscopy^14,21,22^. One could thus anticipate that trifluoroacetate-protein and peptide complexes could be measured the same way as the standard dry-film approach frequently employed in FTIR spectroscopy^23,24^. However, the use of dry-films treated with TFA for FTIR monitoring of proteolytic reactions has not been studied or evaluated in detail.

In the present study, the potential of using FTIR spectra of trifluoroacetate-protein and peptide complexes for gaining information related to proteolytic reactions is explored. FTIR measurements were performed on both TFA-treated and untreated dry-films obtained from different types of protein hydrolysates. Hydrolysis time-series were obtained of a pure protein and more complex raw materials such as by-products from chicken and turkey processing. A thorough evaluation of the approach and its possibilities, together with tentative FTIR band assignments, was also performed. To the best of our knowledge this is the first time TFA is used for modifying protein hydrolysate samples for dry-film FTIR analysis in order to monitor proteolytic reactions.

## Results and Discussion

A range of enzymatic protein hydrolysis reactions were carried out in order to study TFA-induced effects in the dry-film FTIR spectra of the hydrolysate products. The samples were prepared by hydrolysing bovine serum albumin (BSA) and poultry-based raw materials. An overview of the hydrolysate samples and all processing parameters is provided in Table 1. To confirm time-dependent enzymatic degradation, SDS-PAGE electrophoresis and size exclusion chromatography were carried out. These results are presented in the supplementary information (SI) Fig. S1 and Table S1.

**Table 1:** An overview of samples and hydrolysis reaction conditions.

Sample name^a^	Enzyme^b^	Enzyme loading (%)^c^	Reaction time (min)	Water (mL)^d^	Samples per hydrolysis^e^	Raw material (g)^f^	Number of samples
BSAM	Maxipro AFP	4	80	100	12	5	24
CCA	Alcalase	1	80	1000	12	500	24
CCC	Corolase 2TS	1	80	1000	12	500	24
CCF	Flavourzyme	1	80	1000	12	500	24
TMDRA	Alcalase	1	80	1000	12	500	24
TMDRC	Corolase 2TS	1	80	1000	12	500	24
TMDRF	Flavourzyme	1	80	1000	12	500	24
Total							168

^a^Bovine serum albumin (BSA), turkey mechanical deboning residue (TMDR) and chicken carcass (CC). The abbreviations for the enzymes used are added to the raw material  names. ^b^Alcalase (A), Flavourzyme (F), Maxipro AFP (M), and Corolase 2TS (C). ^c^Enzyme loading to raw material. ^d^Water added to reaction mixture. ^e^Number of sampling time points for each reaction. ^f^Raw material loading.

The idea of treating dry-films of protein hydrolysates with TFA prior to FTIR analysis is based on the unique properties of TFA. By adding a large excess of TFA to protein hydrolysate samples, the possible protonation sites of the proteins and peptides will be saturated. This includes the oxygens of the secondary amides in the protein backbone^25,26^. The resulting protein denaturation and protonation will occur using any strong acid. However, TFA has a low boiling point when protonated and also complex-forming abilities. When forming TFA-treated dry-films of hydrolysates, the excess TFA will evaporate and the deprotonated acid (CF_3_COO^−^) will interact as a counter ion with the positive charges on the sample material^27,28^. During an EPH reaction the ratio of N-terminals relative to peptide bonds (secondary amides) is increasing. This ratio will be proportional to the CF_3_COO^−^ complexes formed with N-terminals and secondary amides after the excess TFA evaporation. Therefore, as the proteolytic reaction proceeds, systematic changes are expected in both the CF_3_COO^−^ and amide absorption bands of the denatured proteins and peptides in the corresponding FTIR spectra.

### Spectral changes in TFA-treated dry-films of BSA

Formation of TFA-treated dry-films of hydrolysates was optimised by investigating different concentrations of TFA and other solvent compositions. The simplest and best method was to deposit the sample on the well-plate and let it dry for a minimum of 30 minutes before FTIR measurements. After the first FTIR measurement, a TFA solution was added to the sample. The sample was gently mixed and allowed to dry for a minimum of 30 minutes and measured again.

**Figure 1:**
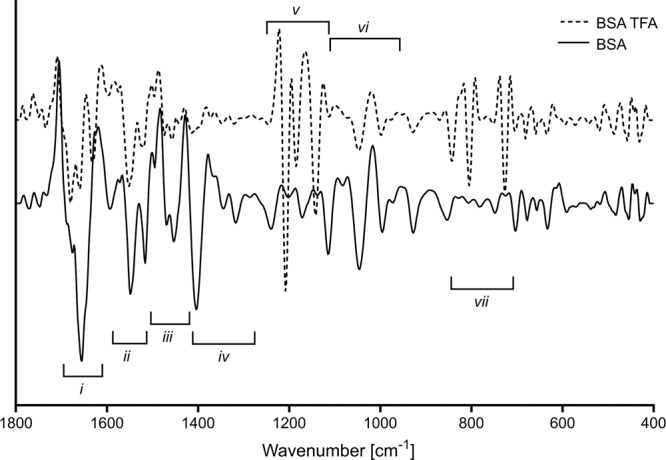
TFA-treated dry-film spectra compared to non-treated spectra of BSA hydrolysed with Maxipro AFP for 80 minutes. Wavenumbers for tentatively assigned FTIR bands are listed in Table 2.

The second derivative FTIR spectrum of BSA hydrolysed with the enzyme Maxipro AFP for 80 minutes is provided in Fig. 1. The spectrum is divided into regions marked *i*-*vii*, and a list with tentative band assignments and wavenumber values is presented in Table 2. The assignments are based on previously published FTIR studies of proteins and peptides^29,30^. Some of these bands have been shown to be important for monitoring proteolytic reactions. These include bands such as the amide I (~1700–1600 cm^−1^) and II (~1590–1520 cm^−1^), the N-terminal (NH_3_^+^, ~1510 cm^−1^) and the C-terminal (COO^−^, ~1400 cm^−1^), but changes can also be observed in other FTIR bands. Figure 1 also shows the FTIR spectrum of the same hydrolysate sample treated with TFA. The formation of the trifluoroacetate-protein or peptide complexes is expected to significantly affect the FTIR spectrum and this was observed by changes in all the defined spectral regions *i*-*vii*. In the regions *i*-*iii*, for example, alterations are seen in all the bands listed above. When comparing the TFA-treated spectrum to the untreated, some of the features of the amide I (~1700–1600 cm^−1^) and II (~1590–1520 cm^−1^) have been preserved whereas the COO^−^ stretches from the peptides and the free amino acids have disappeared altogether due to protonation. New bands from the CF_3_COO^−^ of the trifluoroacetate-protein or peptide complexes are also clearly visible and the most dominant are the C = O (~1677 cm^−1^), C–F (~1250–1100 cm^−1^) and OCO (~950–700 cm^−1^) stretching bands. Protonation of secondary amides are known to affect the amide II (~1590–1520 cm^−1^) FTIR band, as it changes the C-N and N-H bond length of the corresponding amide group^31^. The C-N bond becomes shorter when the carbonyl oxygen is protonated^25^. This gives the C-N bond a stronger double bond nature and the nitrogen will have a positive charge which is stabilised by the CF_3_COO^−^ counter ion. The CF_3_COO^−^ interaction with secondary amide has previously been assigned to an absorption band in the ~1620 cm^−1^ region of the FTIR spectrum. This band has been referred to as diagnostic for the presence of CF_3_COO^−^, and in this study the band is tentatively assigned to an absorption at ~1627 cm^−1^^28^.

**Table 2:** Second derivative bands between 1800–400 cm^−1^ for BSA samples. The tentative assignments are based on literature values^29,41^.

Annotations	Region	Band position cm^-1^	
		BSA	TFA-treated BSA
C = O amide I: turns	*i*	1687	—
C = O (TFA)		—	1677
C = O amide I: α-helix		1656	1656
CF_3_COO^−^		—	1627
COO^−^ (asym stretch)	*ii*	1587	—
Amide II: α-helix		1548	1548
NH_3_^+^ (scissor)	*iii*	1516	1517
CF_3_COO^−^		—	1471
CH_2_ (scissor)		1454	1454
COO^−^ (sym stretch)	*iv*	1404	—
Amide III, CH_2_ (def, rock), OH (def, bend)		1317	—
Amide III, C–O (stretch)		1240	—
C–F (TFA)	*v*	—	1205
C–F (TFA)		—	1182
C–F (TFA)		—	1139
CNH_3_ (rock), CH_2_ (wag)	*vi*	1112	1116
CO, CC, CN (stretch)		1045	1143
CCOO (wagging)		995	995
CH_2_ (twist)		927	925
C–C (TFA)	*vii*	—	840
OCO (TFA)		—	802
OCO (TFA)		—	723

**Figure 2:**
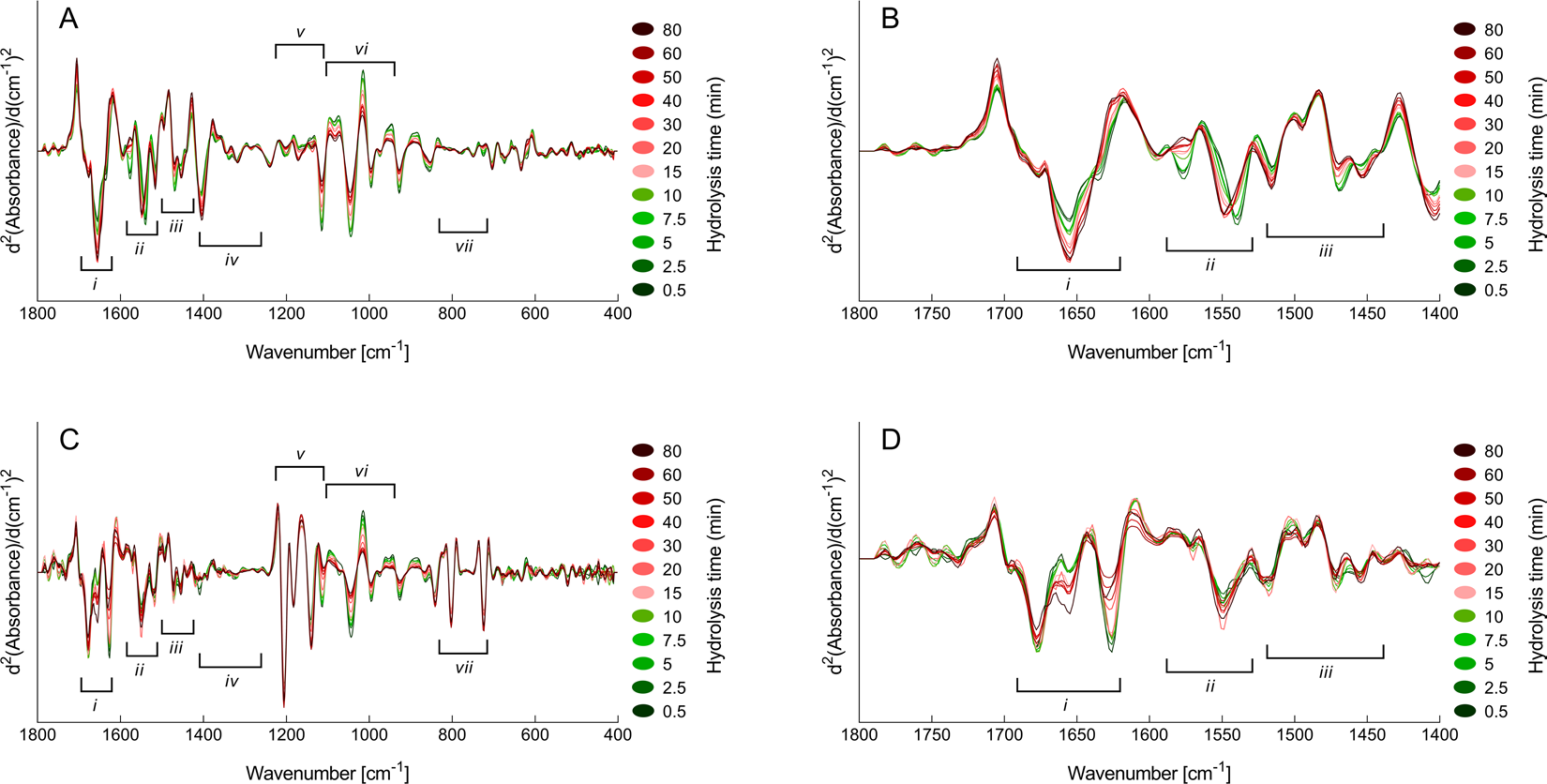
Second derivative FTIR spectra of BSA hydrolysis time-series: (**A**) The spectral region 1800–400 cm^−1^ of the untreated dry-films. (**B**) The spectral region 1800–1400 cm^−1^ of the untreated dry-films. (**C**) The spectral region 1800–400 cm^−1^ of the TFA-treated dry-films. (**D)** The spectral region 1800–1400 cm^−1^ of the TFA-treated dry-films.

Second derivative FTIR spectra of full time-series of BSA hydrolysed with Maxipro AFP are provided in Fig. 2. The untreated FTIR spectra (Fig. 2A) display features closely resembling previously published FTIR spectra of hydrolysates produced using the same protein^29^. By comparing Fig. 2A,C, differences can clearly be observed between the TFA-treated and untreated dry-film spectra. Most important, both time-series show systematic time-dependent changes. For instance, for both time-series, although the spectral changes appearing are different, the amide I (~1700–1600 cm^−1^) band is changing systematically with time. It is also important to note that the new bands appearing in the FTIR spectra of the TFA-treated samples are also systematically changing with time. Examples can be seen by following the C = O (~1677 cm^−1^), CF_3_COO^−^ (~1627 cm^−1^), C–F (~1205 cm^−1^) and the OCO (~802 cm^−1^) bands. The bands assigned to the CF_3_COO^−^ acid group are expected to change as the ratio of N-terminal ends relative to peptide bonds is increasing during the EPH reaction. Finally, the figure clearly reveals that for the major bands in the region 1100–900 cm^−1^, the time-dependent behaviour of the spectra is similar for both untreated and TFA-treated samples. In this region, functional groups with lower polarity give rise to several bands (see Table 2). TFA treatment is therefore expected to provide minimal influence on these bands.

### Spectral changes in TFA-treated dry-films of complex samples

**Figure 3:**
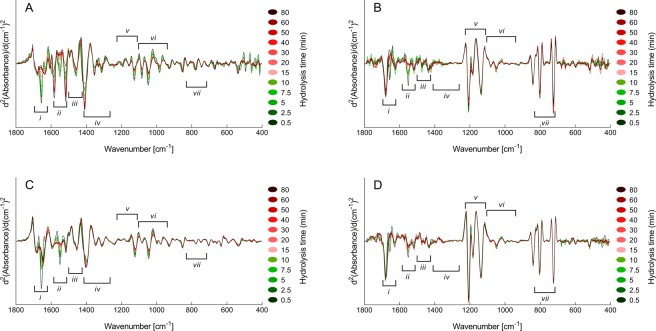
Second derivative FTIR spectra from 1800–400 cm^−1^ of two different hydrolysis time-series, one treated with TFA and one untreated: (**A,B**) Turkey mechanically deboned residue hydrolysed with Flavourzyme. (**C,D**) Chicken carcass hydrolysed with Alcalase. (**A,C**) Untreated dry-film spectra. (**B,D**) TFA-treated dry-film spectra.

The BSA hydrolysates are relatively simple as they originate from a pure protein. The protein is also fairly small and its structure is mostly composed of α-helixes and turns^32^. Time-series of samples obtained from poultry-based raw materials were therefore produced to study the effect of TFA on more complex structures, before and after TFA treatment. Figure 3A,C show the second derivative FTIR spectra of two EPH time-series of poultry substrates. The spectra from the two time-series are visually different from one another and the largest differences are seen in regions *i*-*iii*, where secondary structure attributes are located. These regions also contain important IR bands for EPH monitoring, e.g. amide I (~1700–1600 cm^−1^) and II (~1590–1520 cm^−1^) bands, the N-terminal (NH_3_^+^, ~1510 cm^−1^) and the C-terminal (COO^−^, ~1400 cm^−1^). The complexity difference reflected in the spectra, especially in the amide I and II bands, derives from both the specific raw materials and differences in the mode of action of the specific protease. For example, turkey raw material contains more collagen than the chicken raw material. Also, Flavourzyme (an exopeptidase) is less efficient when it comes to degradation of both muscle proteins and collagens as compared to Alcalase (with mostly endopeptidase activity)^33^. Differences like the ones shown in Fig. 3A,C are typical for hydrolysates originating from different raw materials and enzymes. The spectra of the samples treated with TFA are shown in Fig. 3B,D. Compared to the spectra of the untreated samples, the differences between the spectra of the TFA-treated samples are dramatically reduced (i.e., Fig. 3A vs. 3C as compared to Fig. 3B vs. 3D) and the time-series become more similar to one another. The new CF_3_COO^−^ bands are also directly comparable to the ones described for the TFA-treated BSA spectra in Fig. 2.

**Figure 4:**
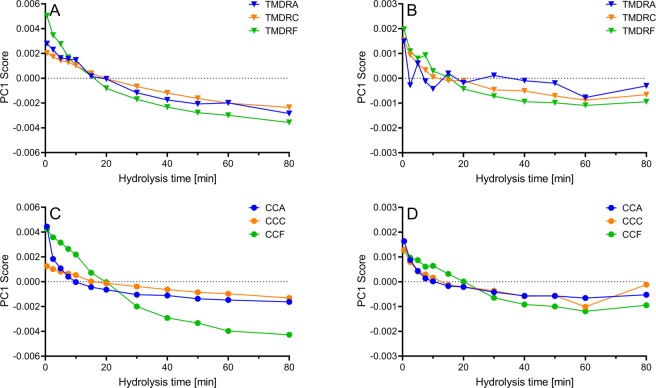
Time-dependent development of PC1 of FTIR spectra during EPH of poultry samples, TFA-treated vs. untreated samples. Sample names and enzyme abbreviations are defined in Table 1. The data points are the average of two time-series. (**A,B**) Turkey mechanically deboned residue. (**C,D**) Chicken carcass. (**A,C**) Untreated FTIR spectra. (**B,D**) TFA-treated FTIR spectra.

The TFA-treated spectra in Fig. 3 show four new bands, C = O (~1677 cm^−1^), CF_3_COO^−^ (~1627 cm^−1^), C–F (~1205 cm^−1^) and OCO (~802 cm^−1^), that seem to change more with increasing hydrolysis time than the others. In order to investigate if these changes contained quantitative information related to the EPH reactions, principal component analysis (PCA) was applied to the FTIR spectra of the EPH time-series. This was done following the description given by Böcker *et al*.^29^. In that study, first component (PC1) scores of each raw material enzyme time-series were seen to depict the change in FTIR signature as a function of hydrolysis time, and it was concluded that the spectral fingerprint of the product is likely to be strongly connected to the composition and homogeneity of the raw material. In the present study each raw material enzyme group consisting of two time-series was subjected to PCA analysis. The PC1 explained 73–93% of the variance for the six untreated groups. The explained variances (PC1) for all groups are presented in SI Table S2. For the TFA-treated samples, 53–70% variance was explained by PC1. Thus, the explained variance was slightly lower for the TFA-treated samples. When plotting the scores of the first component against time as shown in Fig. 4, it can be seen that time-dependent EPH information is present in the spectra time-series of both TFA-treated and untreated samples.

**Figure 5:**
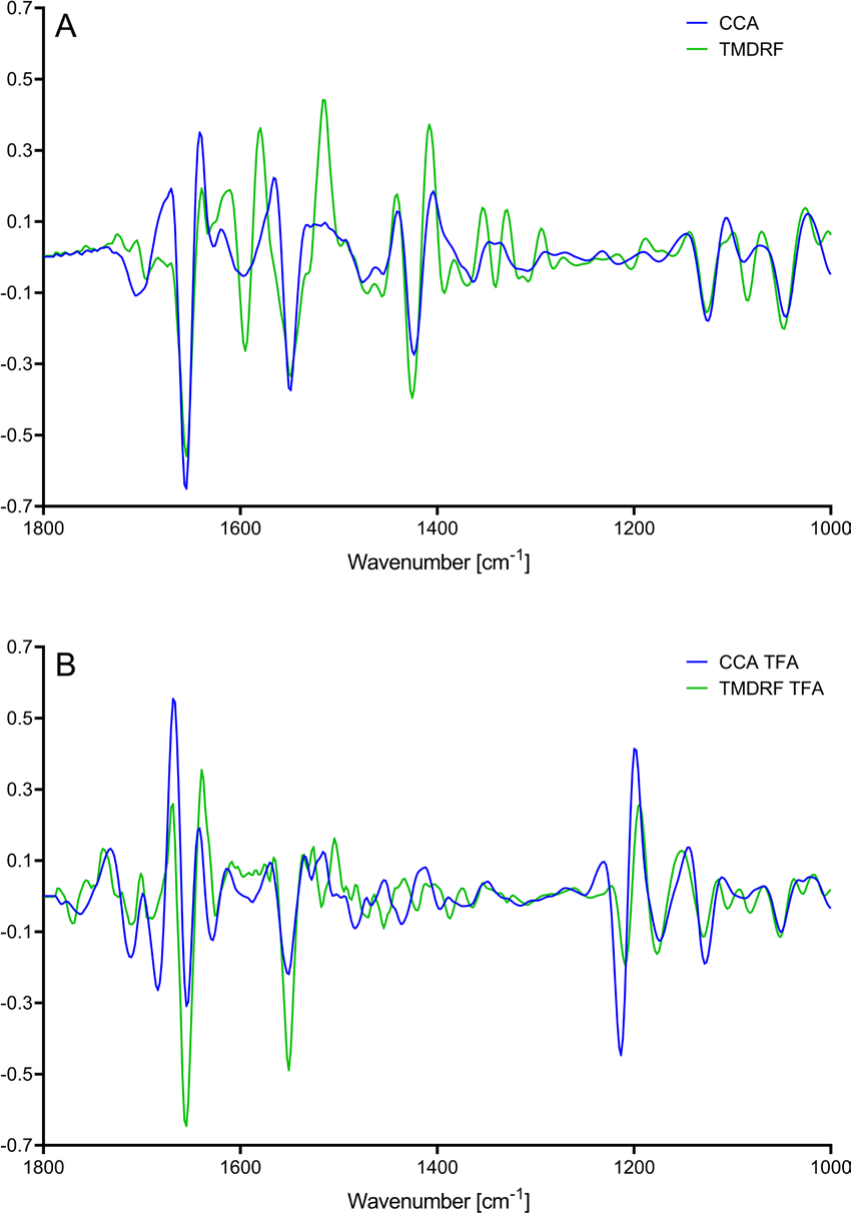
Range-normalised PC1 loadings from 1800–1000 cm^−1^ for the EPH time-series presented in Fig. 3, TFA-treated samples vs. untreated. (**A**) Untreated samples. (**B**) TFA-treated samples.

From the PC1 loadings (Fig. 5) for the different EPH time-series in Fig. 4 it is clear that the FTIR bands important to explain the variation across PC1 changed upon addition of TFA. Figure 5A (i.e. untreated samples) shows that the PC1 loadings of the selected raw material-enzyme combinations are very different in the amide I and II (~1700–1520 cm^−1^) region. Here, information concerning protein secondary structure attributes is located. In the PC1 loadings for the TFA-treated samples (Fig. 5B), on the other hand, one can see that even though there are clear intensity differences in the amide region between the two loadings, the shape of the loadings is very similar. It is also clear that three new peaks related to TFA interactions are important for the spectral variation. These include C = O (~1677 cm^−1^), CF_3_COO^−^ (~1627 cm^−1^), and the C–F (~1205 cm^−1^) bands. Overall, these differences in the loadings indicate that, whereas untreated FTIR samples reveal protein degradation patterns related to raw material specific changes in secondary structures, TFA-treated samples reveal protein degradation patterns related to a more generic CF_3_COO^−^ counter ion effect with secondary amide relative to N-terminal interactions.

### General discussion

The results of this study illustrate the potential of treating dry-films of protein hydrolysates with TFA to reduce spectral differences in FTIR spectra originating from raw material specific protein and peptide features in protein hydrolysates. Currently, these spectral differences are the major factor responsible for the need of large data libraries when samples of different raw material origin are used for prediction of quality parameters such as average molecular weights from FTIR spectra^10^. This study also shows that information important for monitoring proteolytic reactions is preserved in the FTIR spectra of the TFA-treated samples. Thus, it is shown that FTIR spectra of TFA-treated samples contain semi-quantitative information related to protein degradation. The next step is, therefore, to show that FTIR spectra of TFA-treated samples can be used quantitatively.

It is important to remember that the protein hydrolysates studied here, are not pure compounds, but a mixture of thousands of different protein fragments, peptides, amino acids and other water soluble components (e.g. minerals). Specific band assignments of such complex biological systems are very complicated. All band assignments in the present study are thus based on published literature. In addition, a majority of published studies on protein characterisation is related to protein secondary structure. For the protein hydrolysates in the present study it is uncertain how much secondary structure there is left, as the samples are both enzymatically degraded and heat treated to inactivate the enzymes. A thorough study on FTIR band assignments will therefore be needed if FTIR on TFA-treated dry-films is going to be developed as a standardised tool for protein hydrolysate characterisation.

A concern with the TFA treatment is that essential information in the amide I (~1700–1600 cm^−1^) band would be lost under the C = O (~1677 cm^−1^) stretching band from CF_3_COO^−1^^10,21,34^. This is not an uncommon issue in IR spectroscopy. For instance, it can be seen in a study by Poulsen *et al*. predicting DH% in aqueous whey protein hydrolysate samples. Here, the water peak overlaps the amide I band and they concluded that information for monitoring the EPH reaction was partially lost^35^. The difference to this study is that the CF_3_COO^−^ peaks overlapping with the amide I bands are directly linked to the progression of the EPH reaction, as more and more N-terminals are formed. This strengthens the argument that TFA-induced reduction of spectral differences can be utilised for EPH monitoring applications, especially in processes where a variety of raw materials are included.

There are some challenges that have to be overcome in order to fully take advantage of the spectral changes induced by TFA. One of these challenges is dry-film quality. From our observations, the TFA-treated films have a less uniform surface as compared to the untreated ones. This can result in more noise in the FTIR spectra, which in turn can reduce the amount of information available in the spectra. From Figs. 2 and 3 the amount of noise seems to be generally higher in the treated spectra compared to the untreated. To overcome this challenge, we will address several possible solutions to get uniform surfaces of TFA-treated samples in future studies. From printing biomolecules on surfaces for diagnostic applications it is well-known that an appropriate buffer composition and the addition of a surfactant/detergent to the sample may result in uniform surfaces^36,37^. Changing the hydrophobicity of the Si-plate or the relative humidity upon drying of the samples on the plate may be other ways to solve this issue^38,39^.

The FTIR spectra were pre-processed prior to PCA analysis in the current study, as it is generally known that the film-thickness cannot be fully reproduced when performing dry-film FTIR spectroscopy. Thicker films will lead to higher overall absorbance intensities than thinner films, and manual pipetting of small sample aliquots is very difficult to reproduce. Usually, some kind of normalisation procedure like the EMSC approach used in the present study is used to account for this effect^40^. However, if the spectra are very similar, the normalisation procedure will lead to correction of more than the thickness effect. Since TFA treatment could provide such similar spectra, this might be a challenge when quantitative studies are being made. Using internal bands as internal standards, or even adding an external standard, could be possible solutions to this challenge. Alternatively, solutions proposed above to improve spot uniformity may result in a more reproducible spot thickness.

EPH is a growing industrial segment related to valorisation of protein-rich co-streams from the meat and fishery industry as well as others. For these sectors there is a need for simple and fast industrially applicable tools for monitoring product quality development during processing to ensure a constant quality. The present study clearly shows that TFA-treated dry-film FTIR has the potential to simplify the spectra by reducing spectral differences. Consequently, large datasets to build robust quality prediction models based on FTIR spectroscopy will not be necessary anymore. Thus, the technique is a potential future tool for analysis of all types of proteolytic reactions in the laboratory as well as in the industry.

## Conclusion

The present study shows the potential of using dry-films treated with TFA for monitoring changes in proteins and peptides during proteolytic reactions. When TFA interacts with proteins and peptides, complexes are formed that can be dried into films. We show that these complexes contains information important for monitoring proteolytic reactions. Compared to untreated dry-films, where protein degradation patterns are related to raw material specific changes in secondary structures, TFA-treated samples reveal protein degradation patterns related to a more generic CF_3_COO^−^ counter ion effect. There are, however, challenges concerning the dry-film quality and pre-processing of FTIR spectra that have to be investigated and improved in order to fully take advantage of the TFA interactions. When these challenges are solved, FTIR of TFA-treated protein hydrolysates may enable the use of simpler and more generic models for the prediction of quality parameters of proteolytic reactions in laboratory as well as industrial applications.

## Materials and methods

### Materials

Protease from *Bacillus licheniformis* (Alcalase, 2.4 U/g), *Aspergillus oryzae* (Flavourzyme), bovine serum albumin pH 5.2, ≥96% and trifluoroacetic acid were purchased from Sigma-Aldrich (St. Louis, MO, USA). Corolase 2TS was provided by AB Enzyme (Darmstadt, Germany) and Maxipro AFP, by DSM (Delft, the Netherlands). Millex-HV PVDF 0.45 μm 33 mm filters were used for sample preparation (MilliporeSigma, Burlington, MA, USA).

### Raw materials

Raw materials derived from chicken and turkey were hydrolysed by a selection of commercially available enzymes. The poultry raw materials, turkey mechanical deboning residue (TMDR) and chicken carcass (CC) were supplied from a Norwegian slaughterhouse. All samples were minced and packed in bags shortly after arrival, before being stored at −20 °C.

### Enzymatic hydrolysis and sampling

Hydrolysis samples produced using raw materials from poultry were hydrolysed according to the description by Wubshet *et al*.^11^. The BSA hydrolysates were produced by dissolving 5% (w/v) substrate of BSA in tap water. In a screw-cap glass bottle with a magnetic stirrer, 100 mL of the substrate solution was heated to 50 °C in a water bath. When the desired temperature was obtained, 4% (v/w) Maxipro AFP was added to start the enzymatic reaction. From the reaction mixture, 5 mL of sample solution was collected at 0.5 min, 2.5 min, 5 min, 7.5 min, 10 min, 15 min, 20 min, 30 min, 40 min, 50 min, 60 min, and 80 min in 15 mL Falcon tubes. The samples were immediately heated to 90 °C for 10 min to inactivate the enzyme. After cooling to room temperature, the samples were centrifuged (4400 rpm, 15 min) and the clear water phase was collected for freeze drying. The dried samples were dissolved in water to a concentration of 25 mg/mL and filtrated through Millex-HV PVDF (MilliporeSigma, Burlington, MA, USA) before being subjected to spectroscopic analysis. The samples were analysed to verify that the proteins were sufficiently broken down during the EPH reactions. An overview of the samples are given in Table 1.

### FTIR spectroscopy and TFA treatment

From each of the filtered protein hydrolysates, aliquots (7.5 μL) were deposited on 96-well IR-transparent Si-plates and dried at room temperature for at least 30 minutes to form dry films as described by Böcker *et al*.^29^. From each hydrolysate sample, five aliquots were deposited to allow for replicate measurements. FTIR measurements were performed using a High Throughput Screening eXTension (HTS-XT) unit coupled to a Tensor 27 spectrometer (Bruker, Billerica, MA, USA). The spectra were recorded in the region between 4000 and 400 cm^−1^ with a spectral resolution of 4 cm^−1^ and an aperture of 5.0 mm. For each spectrum, 40 interferograms were collected and averaged. Data acquisition was controlled using OPUS v6.5 (Bruker, Billerica, MA, USA). After the first measurements a large excess of TFA (10 μL, 0.25 M TFA in water) was deposited onto each sample well and the slurry was gently stirred with the pipette tip. The plates were then once again dried at room temperature for at least 30 minutes to allow the excess of TFA to evaporate and form dry films before being subjected to FTIR measurements ones again.

### Data treatment

Pre-processing of FTIR spectra from 1800–400 cm^−1^ was performed using Savitzky-Golay second derivative (window size 13 points) followed by extended multiplicative signal correction (EMSC) with second order polynomial and using the mean spectrum as reference. This pre-processing approach enabled removal of major scattering effects in the spectra while at the same time removing the effect of varying thickness of the dry-films^40^. For all subsequent data analysis, the region from 1800–400 cm^−1^ was used. PCA was used for data exploration. Data processing and analysis were carried out using The Unscrambler X v10.3 (CAMO Software AS, Oslo, Norway).

## Supplementary information

Supplementary Information.
