# Supplementary material for: Fourier-transform infrared spectroscopy for monitoring proteolytic reactions using dry-films treated with trifluoroacetic acid
Source: Sci Rep. 2020 May 12;10:7844. doi: 10.1038/s41598-020-64583-3 (PMC7217958; doi:10.1038/s41598-020-64583-3)
Supplement: Supplementary file 1 — Supplementary Information. [file 41598_2020_64583_MOESM1_ESM.docx]

**Supplementary Information**

Fourier-transform infrared spectroscopy for monitoring proteolytic reactions using dry-films treated with trifluoroacetic acid

Kenneth Aase Kristoffersen^a,b*^, Aart van Amerongen^c^, Ulrike Böcker^a^, Diana Lindberg^a^, Sileshi Gizachew Wubshet^a^, Heleen de Vogel-van den Bosch^c^, Svein Jarle Horn^b^ and Nils Kristian Afseth^a^

a) Nofima - Norwegian Institute of Food, Fisheries and Aquaculture Research, P.O. Box 210 N-1431 Ås, Norway. b) Faculty of Chemistry, Biotechnology and Food Science, Norwegian University of Life Sciences (NMBU), P.O. Box 5003, N-1432 Ås, Norway. c) BioSensing & Diagnostics, Wageningen Food & Biobased Research, Wageningen University & Research, Bornse Weilanden 9, 6708 WG, Wageningen, the Netherlands.

** Corresponding author: Kenneth Aase Kristoffersen, kenneth.kristoffersen@nofima.no*

This document contains the results of SDS-PAGE electrophoresis analysis of a selection of BSA time-series samples, the weight-average molecular weight of all the poultry samples studied and the explained variance in PC1 from the PCA analysis.

For the SDS PAGE (Fig. S-1), samples representing a time series of the BSA hydrolysates (2.5, 7.5, 15, 30, 50, 80 min, all 25 mg/ml) was diluted 1:1 with a 2x sample buffer (0.125 M Tris pH 6.8, 4% SDS, 20% glycerol, 0.2 M DTT, 0.04% bromophenol blue) before heating at 10 min at 50⁰C. After cooling, 10 µl of the samples was added to an Invitrogen NuPage Mini 10% Bis-Tris gel using a MOPS running buffer during electrophoresis, performed according to the manufacturer protocol using the XCell *SureLock* Mini-Cell system. The gels were stained with a Coomassie staining solution (1% Coomassie Brilliant Blue R, 40% MeOH, 7% acetic acid) for one hour, and destained overnight (40% MeOH, 7% acetic acid in distilled water). The weight-average molecular weights (Table S-1) were measured using the method described by Wubshet et al.^1^


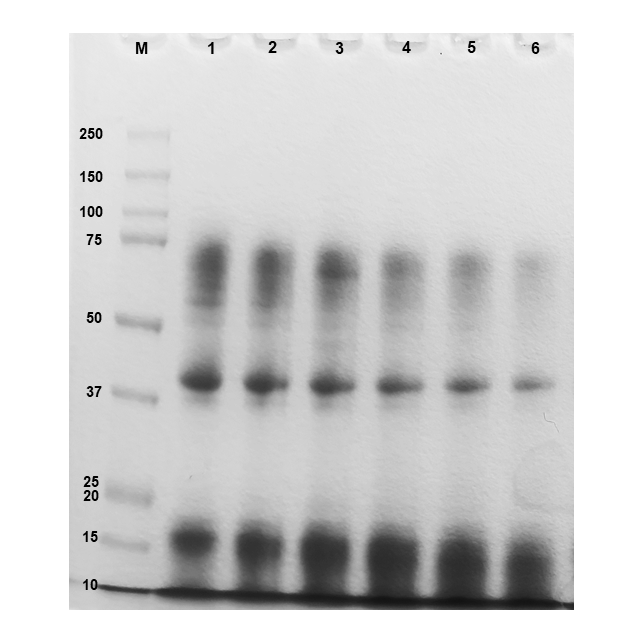


**Fig S-1**: The resulting SDS-PAGE gel. In lane M, the marker with the molecular weights defined in the BioRad Dual color prestained protein ladder product sheet, and to the left in the figure. In lane 1 to 6, the hydrolysates from 2.5, 7.5, 15, 30, 50, 80 min.

**Table S-1:** Weight-average molecular weight (*M*_w_, g/mol) values for all poultry EPH samples.

| Time (min)^a^ | 1) CCA | 1) CCC | 1) CCF | 1) TMDRA | 1) TMDRC | 1) TMDRF |
| --- | --- | --- | --- | --- | --- | --- |
| **0.5** | 3246.3 | 2558.5 | 1617.1 | 3972.8 | 2595.9 | 3845.7 |
| **2.5** | 2796.8 | 2463.9 | 1653.5 | 5512.9 | 2780.4 | 3083.7 |
| **5** | 2714.1 | 2608.1 | 1821.6 | 3104.3 | 2816.5 | 6493.1 |
| **7.5** | 2702.4 | 2548.6 | 1621.1 | 5076.7 | 2706.8 | 2714.4 |
| **10** | 2624.4 | 2440.0 | 2629.5 | 2580.5 | 2845.3 | 2583.1 |
| **15** | 2288.1 | 2383.2 | 1557.8 | 4692.5 | 2658.2 | 4204.7 |
| **20** | 2208.3 | 2226.4 | 2669.8 | 2441.4 | 3001.2 | 2253.9 |
| **30** | 2042.7 | 2256.6 | 1381.6 | 2288.8 | 2649.1 | 2354.8 |
| **40** | 2038.5 | 2392.9 | 1310.6 | 2353.5 | 2649.1 | 1971.0 |
| **50** | 1872.3 | 2017.6 | 1247.8 | 2336.0 | 2366.5 | 1857.7 |
| **60** | 1889.7 | 2000.8 | 1206.2 | 2936.2 | 2383.3 | 1828.6 |
| **80** | 1742.7 | 1902.4 | 1139.7 | 1967.3 | 2320.2 | 1628.6 |
| **Time (min)^b^** | 2) CCA | 2) CCC | 2) CCF | 2) TMDRA | 2) TMDRC | 2) TMDRF |
| **0.5** | 5352.6 | 2394.3 | 1726.7 | 3940.4 | 2611.8 | 4806.0 |
| **2.5** | 2507.5 | 2266.1 | 1909.3 | 4968.4 | 2580.5 | 4129.6 |
| **5** | 2439.4 | 2142.5 | 1921.0 | 4175.9 | 3213.2 | 4574.5 |
| **7.5** | 3290.0 | 2563.8 | 1828.2 | 4897.5 | 2941.3 | 2944.2 |
| **10** | 2232.0 | 2220.6 | 1921.7 | 2503.3 | 2622.3 | 2514.2 |
| **15** | 2125.5 | 2566.7 | 2680.8 | 2291.3 | 2498.5 | 2365.3 |
| **20** | 1949.7 | 2007 | 3345.1 | 3966.9 | 2317.8 | 2201.5 |
| **30** | 1818.9 | 2175.1 | 1629.3 | 2131.1 | 2286.7 | 2065.7 |
| **40** | 2154.6 | 2173.7 | 1553.5 | 1977.1 | 2398.2 | 1934.8 |
| **50** | 1642.4 | 2169.5 | 1483.5 | 1881.7 | 2365.5 | 1810.4 |
| **60** | 1578.6 | 2342.7 | 1379.7 | 1823.1 | 2259.3 | 1757.7 |
| **80** | 1572.7 | 2016.6 | 1310.0 | 1699.2 | 2479.5 | 1640.6 |

a) First replicates b) Second replicates

**Table S-2:** Explained variance in PC1 from PCA analysis for all poultry time-series.

| **PC1** | Untreated (%) | TFA-treated (%) |
| --- | --- | --- |
| **CCA** | 85 | 62 |
| **CCC** | 74 | 70 |
| **CCF** | 93 | 60 |
| **TMDRA** | 73 | 59 |
| **TMDRC** | 81 | 64 |
| **TMDRF** | 80 | 53 |

References

1 Wubshet, S. G. *et al.* FTIR as a rapid tool for monitoring molecular weight distribution during enzymatic protein hydrolysis of food processing by-products. *Analytical Methods* **9**, 4247-4254, doi:10.1039/C7AY00865A (2017).
